# Block Copolymer Synthesis by the Combination of Living Cationic Polymerization and Other Polymerization Methods

**DOI:** 10.3389/fchem.2021.644547

**Published:** 2021-06-28

**Authors:** Asmita Dey, Ujjal Haldar, Priyadarsi De

**Affiliations:** Polymer Research Centre and Centre for Advanced Functional Materials, Department of Chemical Sciences, Indian Institute of Science Education and Research Kolkata, Kolkata, India

**Keywords:** cationic polymerization, block copolymer, macroinitiator, dual initiator, site transformation, polyisobutylene

## Abstract

The foremost limitation of block copolymer synthesis is to polymerize two or more different types of monomers with different reactivity profiles using a single polymerization technique. Controlled living polymerization techniques play a vital role in the preparation of wide range of block copolymers, thus are revolutionary techniques for polymer industry. Polymers with good control over molecular weight, molecular weight distribution, chain-end functionality and architectures can be prepared by these processes. In order to improve the existing applications and create new opportunities to design a new block copolymer system with improved physical and chemical properties, the combination of two different polymerization techniques have tremendous scope. Such kinds of macromolecules may be attended by combination of homopolymerization of different monomers by post-modification techniques using a macroinitiator or by using a dual initiator which allows the combination of two mechanistically distinct techniques. This review focuses on recent advances in synthesis of block copolymers by combination of living cationic polymerization with other polymerization techniques and click chemistry.

## Introduction

Macromolecular engineering is the technology of total synthesis of highly controlled macromolecules, with the goal to achieve control over the physical properties of macromolecules, including molecular weight, molecular weight distribution, end-functionality, tacticity, stereochemistry, block sequence, and block topology. The best technique for the preparation of polymers with well-defined structures having low dispersity is living polymerization. In the living polymerization process, all chains are initiated during the start of the polymerization and the chains grow at a constant rate retaining their chain-end fidelity (Moad et al., 2008; Jenkins et al., 2009). The term living polymerization was first coined by Szwarc et al. (1956), who introduced living anionic polymerizations in 1956 (Szwarc, 1956). Later, the living cationic polymerization, living ring-opening polymerization (ROP) and other living polymerization methods were discovered.

Cationic polymerization is an important technique for polymer synthesis for monomers having an electron-rich double bond, such as, isobutylene (IB) (Rajasekhar et al., 2020). Conventional cationic polymerization progresses through four elementary steps: initiation, propagation, chain transfer, and chain termination. Cationic species are generated by ionization during initiation and electrophilic addition of the monomer to the active cationic site takes place during propagation. The conventional cationic polymerization reactions also involve chain termination, chain transfer, and other chain-breaking reactions. This is due to the high reactivity of the active cationic species. Thus, the propagating active species gets quenched and kinetic chain is stopped. Chain transfer reactions limit the molecular weight; thus, to obtain high molecular weight polymers, reactions are mostly carried out at very low temperature to prevent chain transfer reactions. Polymers bearing selectively placed functional groups at the chain end(s) are also very difficult to synthesize using conventional cationic polymerization techniques. So, living cationic polymerization was introduced to synthesize polymers having controlled molecular weight, with limited chain transfer and termination processes; narrow molecular weight distribution and predetermined chain end functionalities. Living cationic polymerization was first reported using vinyl ethers (Miyamoto et al., 1984) and isobutylene (Faust and Kennedy, 1987) in the 1980s. Since then, the scope of living cationic polymerization has been expanded rapidly both in terms of monomers and initiating systems.

The capability to control the polymerization process opens up a tremendous scope for the synthesis of highly controlled polymeric architectures such as random, alternating, star, branched, block, graft copolymers, etc. Presently, choice and sequence addition of monomers for block copolymerization using a single polymerization process has limitations since there is no single polymerization technique for all types of monomers. There are many reported review articles which have discussed all types of combination techniques, but here our aim is to focus typically on synthetic procedures for copolymers by living cationic polymerization with other polymerization techniques. This review summarizes the recent developments in the synthesis methodologies of various types of polymeric materials by combined cationic polymerization pathways with other polymerization techniques like radical, anionic, ring opening polymerisation, click chemistry, etc.

## Living Cationic Polymerization

**FIGURE 1:**
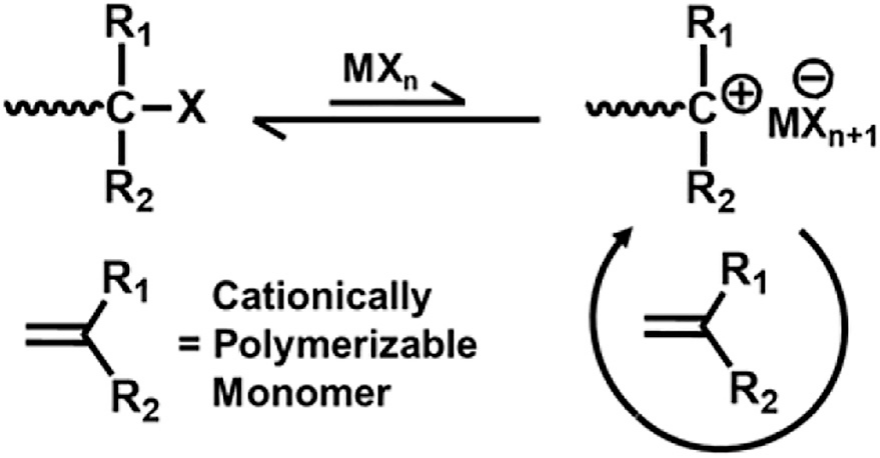
General mechanism of controlled/living cationic polymerization.

The key to achieve living cationic polymerization is controlled initiation and propagation (Aoshima and Kanaoka, 2009). To achieve polymers with controlled molecular weight, narrow polydispersity, and precise chain end functionality, elimination or suppression of chain transfer or chain termination reactions are essential (Cho et al., 1990). Living cationic polymerization of various electron rich vinyl monomers are initiated by Lewis acid containing a binary initiating system to produce a carbocation structurally similar to the monomer (De and Faust, 2007). For living cationic polymerization, the key factor is to attain equilibrium between the formed active species and the dormant polymer chain, and there must be rapid exchange within the active and dormant species so that a small amount of active species is present within the system (Figure 1). Factors like temperature, solvent polarity, concentration of active initiating species, and nature of the counter ion play a decisive role to control the equilibrium of the entire mechanism.

The first report on living cationic polymerization was for isobutyl vinyl ether (IBVE) by Higashimura et al. using HI/I_2_ initiating system (Sawamoto and Higashimura, 1986). Since then, researchers have grown interest in cationic polymerization of other vinyl monomers. Faust and Kennedy (1987) reported living cationic polymerization IB. The scope of living cationic polymerization has increased in terms of initiating systems, monomers, and synthetic applications in the course of time. Linear semilogarithmic kinetic plot (ln([*M*]_0_/[*M*]) vs. time, where [*M*]_0_ is the initial concentration and [*M*] is the concentration at time (*t*) = *t* of the monomer) suggests constant concentration of active species, i.e., no chain termination; and linear dependence of number average molecular weight (*M*_n_) vs. monomer conversion (*M*_n_ vs. conversion) indicates the absence of chain transfer (Szwarc and Beylen, 1993). Again, according to a combined relation derived by Penczek et al. (1991), the linearity of the ln{1-([*I*]*DP*_n_/[*M*]_0_)} vs. time plot [where *DP*_n_ is the number average degree of polymerization, (*I*) is the concentration of the initiator] proves the simultaneous absence of chain termination and chain transfer (Penczek et al., 1991).

Generally, two methods are used for the preparation of functional polymers *via* living cationic polymerizations: (1) functional initiators and (2) end quenching of living polymeric cations with appropriate nucleophiles or capping agents. A functional initiator with/without a protected functional group is generally used during the living cationic polymerization to produce functional polymers. Several reports are there for the preparation of chain-end functional polyisobutulenes (PIBs) using ester functional initiators such as 3,3,5-trimethyl-5-chloro-1-hexyl isobutyrate and methacrylate (Balogh et al., 1992). An efficient way to produce end-functional polymers is by end quenching of living cationic polymerizations with appropriate nucleophiles. The functional initiator method is more efficient due to unwanted side reactions during nucleophile act to the end functionalization of the propagating polymer cations. PIB with functional terminator approach was reported by Faust et al., using diphenylethylene (DPE) (Hadjikyriacou et al., 1995). This strategy was further extended to prepare a variety of end-functional PIBs bearing, alcohol, methoxy, amine, carbonyl, and ester end groups.

The initiator system plays an important role according to monomer choice. A wide variety of initiators were reported such as, organic esters, halides, ethers, and alcohols, which act as cation source to initiate living polymerization of IB. Hydrogen chloride (HCl) adducts of 2-chloro-2,4,4-trimethylpentane and 2-chloro-2,4-diphenyl-4-methylpentane works as excellent initiators for *α*-methyl styrene and IB, respectively. Faust et al., reported weak Lewis acids as co-initiator for more reactive monomers like vinyl ethers or *N*-vinyl carbazole, whereas strong Lewis acids for less reactive monomers like IB or styrene (De and Faust, 2015).

## Combination of Living Cationic Polymerization with Other Polymerization Techniques

**FIGURE 2:**
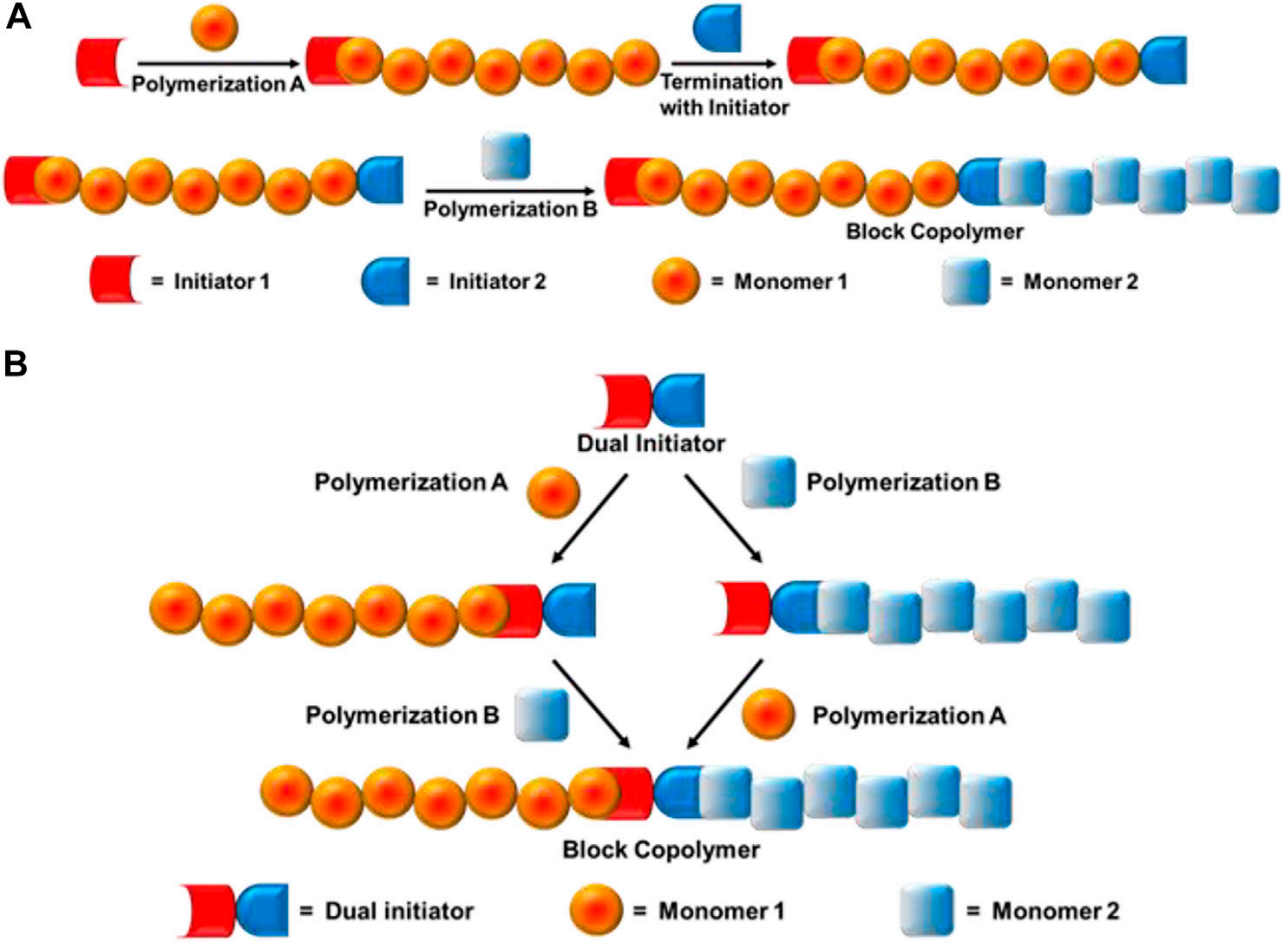
Block copolymer synthesis mechanism: **(A)** Site transformation method, and **(B)** dual initiator method.

Novel block copolymers could be synthesized by combination of different living/controlled polymerization mechanisms such as radical, cationic, anionic, ring-opening polymerizations, click chemistry, etc., with new monomer combinations. The first report on combination polymerization was by Richards et al., in 1977 to solve the problem of synthesizing copolymers using different types of monomers (Burgess et al., 1977a; Burgess et al., 1977b). The combination of two mechanisms occurs mainly by two types of processes: a) site transformation method, and b) dual initiator method (Figure 2). The site transformation technique provides a useful alternative for the synthesis of block copolymers, where the propagating active centre is transformed to a whole new kind of active site and a second monomer is polymerized by a different mechanism using the newly formed active site, i.e., one homopolymer chain is terminated by an initiator which initiates block copolymerization using another technique. Whereas, in the dual initiator method, one single initiator is capable of initiating polymerization in two different mechanisms. Another important technique is by combination of living cationic polymerization with coupling click chemistry. The essential conditions to achieve quantitative coupling reaction is that the end groups should have similar reactivities and selection of a good solvent for both homo—and copolymers.

### Combination of Living Cationic Polymerization with Controlled/Living Radical Polymerization

From the very first introduction of the combination polymerization technique concept, researchers’ interest in the synthesis of novel polymeric architectures has grown. The scope of combination polymerization has got new dimensions by preparation of block copolymers using cationic and controlled/living radical polymerizations like atom transfer radical polymerization (ATRP), reversible addition-fragmentation chain-transfer (RAFT) polymerization, nitroxide-mediated polymerization (NMP), etc.

### Combination with Atom Transfer Radical Polymerization

**FIGURE 3:**
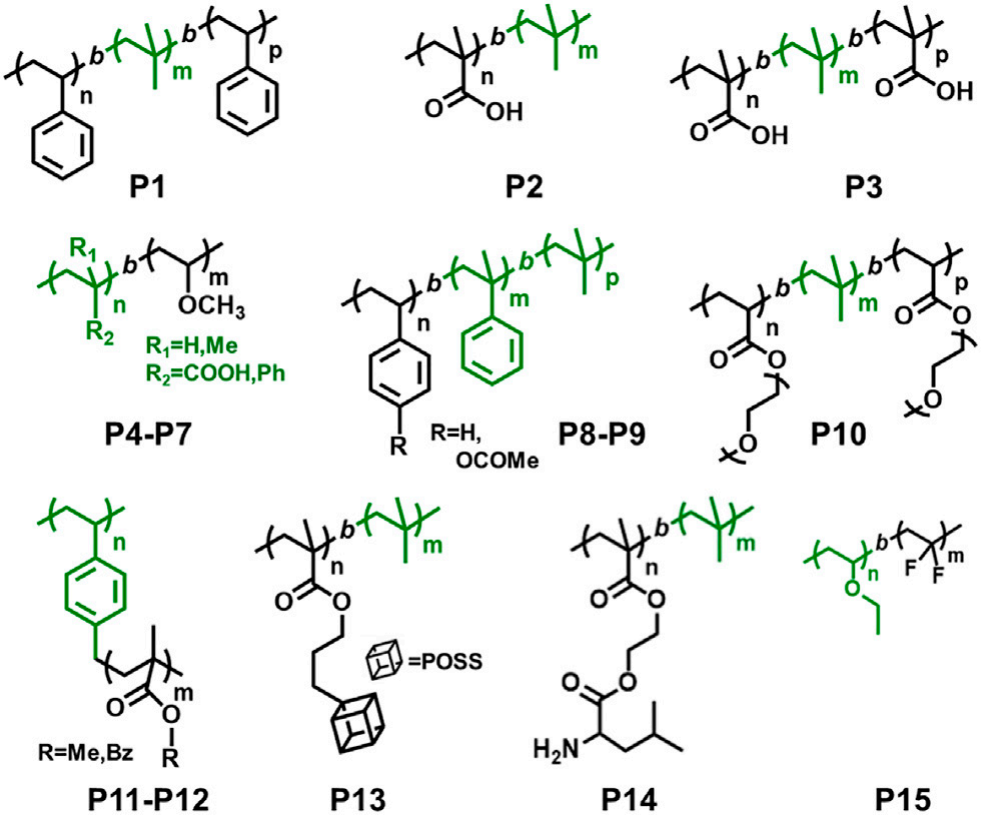
Examples of block copolymers synthesized *via* combination of living cationic polymerization and controlled/living radical polymerization.

**FIGURE 4:**
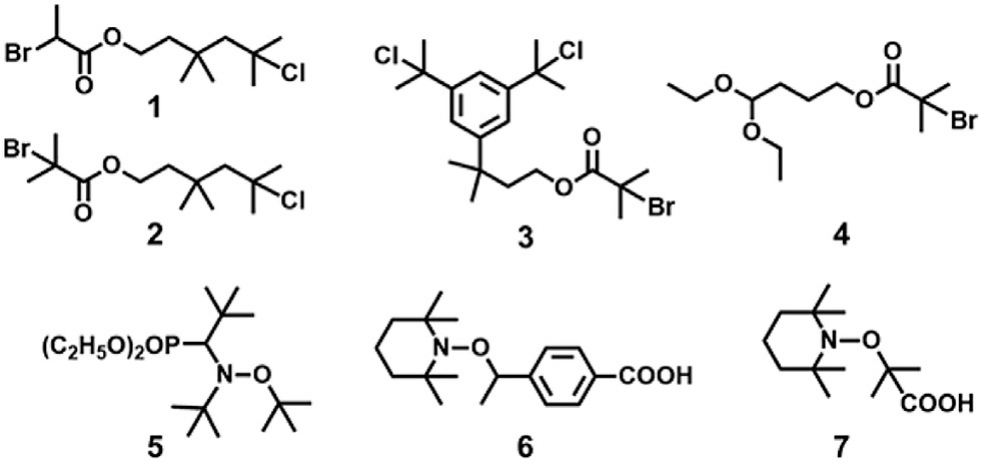
Examples of dual initiators used in combined living cationic polymerization and controlled/living radical polymerization.

In 1997, Coca and Matyjaszewski reported block copolymers preparation of styrene (St) and IB, i.e., P(St-b-IB-b-St) (**P1**) by combination of cationic and ATRP, where both St and IB monomers are capable of undergoing polymerization by living cationic techniques. A recent study proves P(St-b-IB-b-St) (**P1**) is highly hemocompatible and can be used as heart valve leaflets in near future (Table 1) (Ovcharenko et al., 2019). Researchers have also studied surface mobility of heparin by synthesizing functional dendritic PIB-based thermoplastic elastomer by styrene (St) and IB copolymer (Wu et al., 2018). However, the insertion of monomers like methacrylic acid (MA) with the same polymerization approach is a real difficult task as it does not undergo polymerization *via* cationic polymerization technique. Hence, the synthesis of P(IB-*b*-MA) (**P2**) di-block copolymer, P(MA-*b*-IB-*b*-MA) (**P3**) tri-block copolymer is more fascinating (Fang and Kennedy, 2002). They have reported micellar and endless ionomer networks using these triblock copolymers (Table 1). The PIB macroinitiator used for the ATRP was synthesized from the hydroxyl functional PIBs, obtained by hydroboration/oxidation of allyl functional PIBs, by further reaction with 2-bromoisobutyryl bromide. For the synthesis of **P2** and **P3** (Figure 3), *tert*-butyl methacrylic acid (*t*-BuMA) was polymerized using the macroinitiator and then further hydrolysed post polymerization to synthesize the targeted block copolymers. Synthesis of **P2** by dual initiator has also been reported, where dual initiators (Figure 4) like 3,3,5-trimethyl-5-chlorohexyl 2-bromopropionate (IB_2_BP, **1**) and 3,3,5-trimethyl-5-chlorohexyl 2-bromo-2-methylpropionate (IB_2_BMP, **2**) were used to combine cationic polymerization and ATRP (Zhu and Storey, 2010). A similar type of dual initiator, 3-[3,5-bis(1-chloro-1-methyl ethyl)phenyl]-3-methyl butyl-2-bromo-2-methyl propionate (DCCBMP, **3**) was reported by Zhu and Storey (2012) for the synthesis of polyisobutylene-based miktoarm star polymers.

**TABLE 1:** Polymers, their morphologies, and applications.

Polymers	Morphology	Applications	References
P(St-b-IB-b-St) (**P1**)	Micelle	Hemocompatible	Coca and Matyjaszewski (1997)
		Heart valve leaflets	
P(IB-*b*-MA) (**P2**)	Micelle	Thermoplastic elastomer	Fang and Kennedy (2002)
P(MA-*b*-IB-*b*-MA) (**P3**)	Micelle	Thermoplastic elastomer	Fang and Kennedy (2002)
	Ionomer network		
P(PEGMA-*b*-IB-*b*-PEGMA) (**P10**)	Crystalline	Non-ionic surfactant	Szabó et al. (2015)
P(MAPOSS-*b*-IB) (**P13**)	Crystalline	Composite material	Haldar et al. (2015b)
P(NH2-L-Leu-HEMA-*b*-IB) (**P14**)	Micelle	pH triggered delivery	Bauri et al. (2013)
P(ethylene-IB-*b*-ethylene) (**P23**)	–	Thermoplastic elastomer	Espinosa et al. (2013)

**FIGURE 5:**
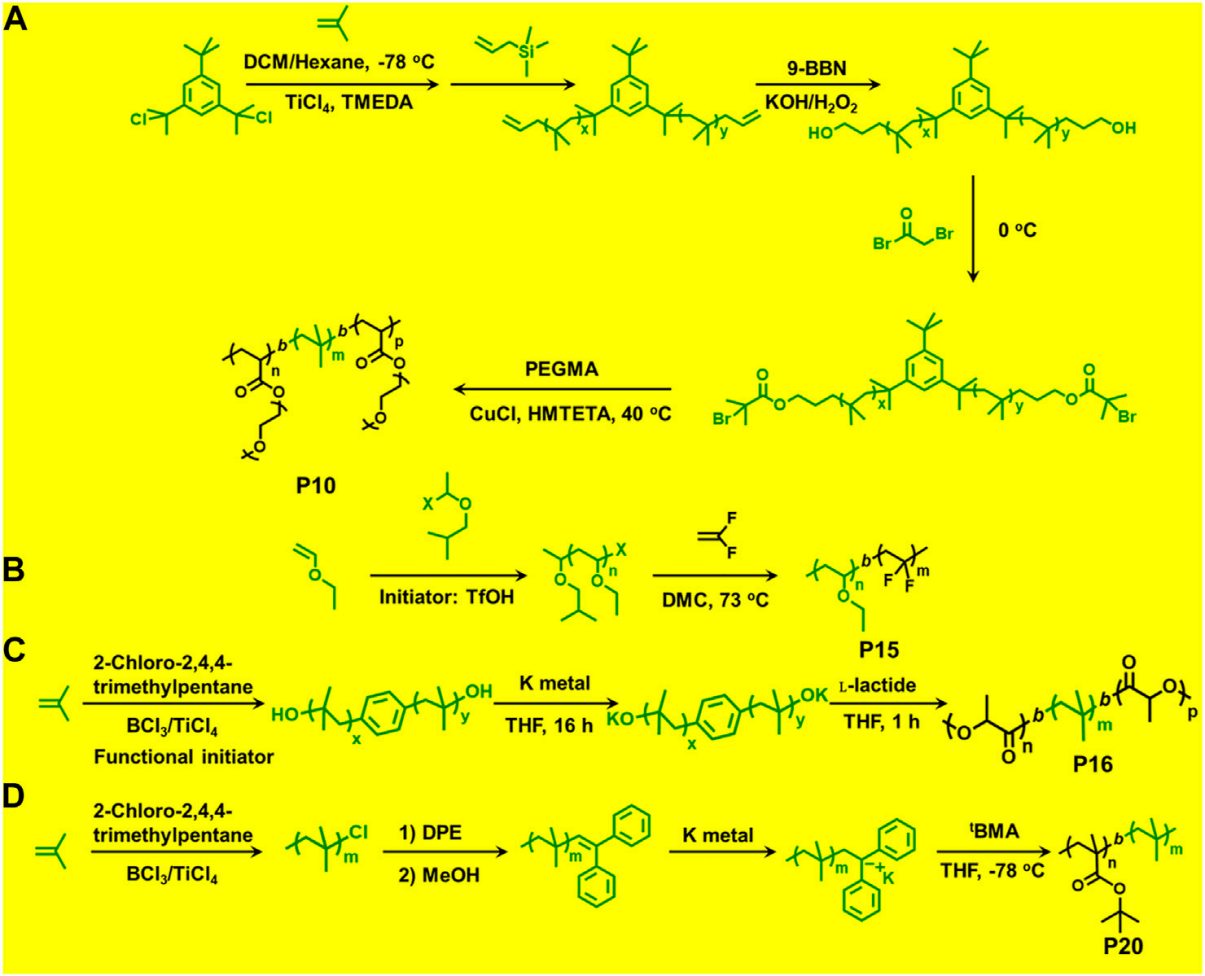
**(A)** Block copolymer synthesis *via* combination of living cationic polymerization and atom transfer radical polymerization. **(B)** Block copolymer synthesis *via* combination of living cationic polymerization and radical polymerization polymerization. **(C)** Block copolymer synthesis *via* combination of living cationic polymerization and ring-opening polymerization. **(D)** Block copolymer synthesis *via* combination of living cationic polymerization and anionic polymerization.

Du Prez et al., prepared block copolymers of poly(methyl vinyl ether) (PMeVE) segment with poly(*tert*-butyl acrylate), poly(acrylic acid), poly(methyl acrylate), and polystyrene blocks (**P4–P7**), using a novel dual initiator 2-bromo-(3,3-diethoxy-propyl)-2-methylpropanoate (**4**) (Bernaerts and Du Prez, 2005). In 2008, P(IB-*b*-St) and poly(IB-*b*-poly(methyl methacrylate-*co*-styrene) P[IB-*b*-(MMA-*co*-St)] copolymers were reported using a combined ATRP process using PIB with allyl halide end groups as macroinitiators (Jakubowski et al., 2008). Chen et al. (1998) reported ATRP of styrene in bulk and in xylene solution and *p*-acetoxystyrene (*p*ACOSt) (**P8-P9**) in xylene solution using 1-chloro-1-phenylethyl-telchelic PIBs (*M*_n_ = 7,800 and 30,700 g/mol) as macroinitiators (Chen et al., 1998). Triblock copolymers (**P10**) composed of hydrophobic inert PIB as an inner core and hydrophilic poly[poly(ethylene glycol) methacrylate] (PPEGMA) outer blocks were reported *via* combined ATRP and cationic polymerization techniques (Figure 5; Szabó et al., 2015). This copolymer having a crystalline domain can be applied as nonionic surfactants (Table 1). Polymerization of bisazobenzene containing vinyl ether by cationic polymerization with IBVE as initiator using Et_1.5_AlCl_1.5_ as catalyst, and ethyl acetate as base was synthesized, which acts as macroinitiator for block copolymerization of methyl methacrylate (MMA) by ATRP (Li et al., 2014). Spherical and worm like polymeric architectures were formed by poly(*p*-chloromethyl styrene)-*g*-(methyl methacrylate) P(*p*CMS-g-MMA) (**P11**) and poly(*p*-chloromethyl styrene)-*g*-(benzyl methacrylate) P(*p*CMS-*g*-BzMA) (**P12**) graft copolymers (Banerjee et al., 2014). Initially, P(*p*CMS) macroinitiator was synthesized using FeCl_3_ at 25°C by cationic polymerization, which in turn initiated the ATRP process for MMA or BzMA monomers. Researchers have reported triblock copolymers having rod-coil-rod morphology with liquid crystal properties (Gao et al., 2008). They form rod and lamellar structures which can be confirmed from TEM images. Some other block copolymer preparation with monomers such as 2,5-bis[(4-methoxy phenyl)oxycarbonyl]styrene (*p*MPCS) using PIB macroinitiator are also reported by ATRP technique. PIB-*b*-oligoacrylates and PIB-*b*-oligomethacrylates were synthesized by ATRP using PIB-*α*-bromo ester macroinitiator (Fu et al., 2018).

*β*-Pinene is another very important monomer for living cationic polymerization. There are numerous reports on copolymers containing *β*-pinene by living cationic technique but few studies are reported by using combination mechanism. Block copolymers with *β*-pinene polymer-based macroinitiator is also well studied. *β*-Pinene macroinitiator was prepared by living cationic polymerization with the 1-phenylethyl chloride/TiCl_4_/Ti(O*i*Pr)_4_/*n*Bu_4_NCl system, and used for block copolymerization of MMA or butyl acrylate (BA) (Lu et al., 2003) and St (Lu et al., 2004).

### Combination with Reversible Addition-Fragmentation Chain-Transfer Polymerization

To date, several researchers have reported preparation of novel block copolymers by combination of living cationic and RAFT polymerizations (Minoda et al., 2013; Sugihara et al., 2012). There are various reports of block and graft copolymers *via* combination of living cationic polymerization and RAFT polymerization using vinyl ether type RAFT agent, such as benzyl 2-(vinyloxy)ethyl carbonotrithioate (BVCT). The BVCT act as dual initiator; it acts as a cationogen under EtAlCl_2_ initiation system in the presence of ethyl acetate for living cationic polymerization and as a RAFT agent for the blocks for RAFT polymerization mechanism (Ma Radzi et al., 2014a). De and co-workers made an effort to prepare a well-defined di-block copolymers of IB with poly[oligo(ethylene glycol) methyl ether methacrylate] (POEGM; Bauri et al., 2015) poly(3-(3,5,7,9,11,13,15-heptaisobutyl-pentacyclo[9.5.1.1^3,9^.1^5,15^.1^7,13^]-octasiloxane-1-yl)propyl methacrylate) (PMAPOSS) (**P13**) (Haldar et al., 2015a) and amino acid-based monomers such as Boc-l-alanine methacryloyloxyethyl ester (Boc-l-Ala-HEMA) and Boc-l-leucine methacryloyloxyethyl ester (Boc-l-Leu-HEMA) (**P14**) *via* combination of living carbocationic and RAFT polymerizations techniques (Bauri et al., 2013). The amino acid-based block copolymers were synthesized using hydroxyl end-capped polyisobutylene as macro chain transfer agents (mCTAs). The side-chain amino acid-based block copolymers showed core-shell type micellar aggregates in methanol, but after the Boc group deprotection, the block copolymers formed spherical micellar aggregates in aqueous milieu. These stable micellar aggregates can be applied in chiral recognition, chiral resolution. They may also be used in chiral catalysis of some organic reactions. Whereas the POSS containing polymers showed crystallinity and are used for synthesis of biocompatible composite materials (Table 1). Self-healing gel was constructed from side-chain primary amine leucine pendant with PIB. The diblock copolymer P(H_2_N-Leu-HEMA)-*b*-IB) (**P14**) was synthesized using PIB based di-functionalized cross-linker (HOC-PIB-CHO) without aiding any external stimuli (Haldar et al., 2015b). They show well defined crosslinking and are self-healing, thus finding a wide range of applications in organ repair and pH-triggered delivery.

Synthesis of poly[(ethyl vinyl ether)-*b*-(vinylidene fluoride)] P(EVE-*b*-VDF) (**P15**) diblock copolymer was reported *via* the sequential combination of cationic polymerization of vinyl ethers and radical RAFT polymerization of vinylidene fluoride (VDF) (Figure 5; Guerre et al., 2017). Metal-free RAFT cationic combination polymerization was reported by Maeda et al. for the synthesis of block copolymers. For synthesis of vinyl ethers (VEs) macroinitiator, the 1-isobutoxyethyl ethane dithioate (IDTA) acts as a catalyst and the cationic polymerization was initiated by the HCl.Et_2_O pair. The resulting poly(vinyl ethers) (PVEs) could be used as mCTA for the RAFT polymerization of methacrylate and styrene (Sugihara et al., 2015). The copolymers show core-shell morphology which has been confirmed from Atomic Force Microscopy (AFM) images.

Novel xanthates containing moieties were designed for synthesis of block copolymer *via* combination of living cationic polymerization and RAFT polymerization. Cationic polymerization of IBVE and *tert*-butyl vinyl ether (*t*BVE) were reported using xanthate containing initiating system. Initially the cationic polymerizations of IBVE and *t*BVE were conducted by using *S*-benzyl *O*-2-(vinyloxy)ethyl carbonodithioate (Xanthate 1)-HCl adduct/SnCl_4_ and Xanthate 1 or *S*-1-(ethoxycarbonyl)ethyl *O*-2-(vinyloxy)ethyl carbonodithioate (Xanthate 2)-CF_3_COOH adduct/EtAlCl_2_ initiating system in the presence of ethyl acetate. Then, RAFT/MADIX polymerization of vinyl acetate (VAc) was carried out using azobis(isobutyronitrile) (AIBN) as initiator at 60°C using either poly(IBVE) or poly(TBVE) macro-CTA. The poly(TBVE) mCTAs synthesized from the Xanthate 2 were able to polymerize VAc *via* RAFT/MADIX polymerization, and produced well-defined diblock copolymer, poly(TBVE)-*b*-poly(VAc) (Ma Radzi et al., 2014b).

### Combination with Nitroxide-Mediated Polymerization

In Le et al. (2016), first reported the block copolymers by combination of NMP and living cationic polymerization. They have made some dual initiators for this particular concern. Among the various dual initiators (**5**–**7** in Figure 4), TEMPO-based-alkoxyamine having high functionality of TEMPO showed high efficiency in controlled polymerization of IBVE. The TEMPO-functionalized poly(IBVE) (PIBVE) was used as a macroinitiator for St at 130°C by NMP, resulting a well-defined block copolymer of PIBVE-*b*-PS having a narrow dispersity (*Ð* = 1.2).

## Combination of Living Cationic Polymerization with Ring Opening Polymerization

**FIGURE 6:**
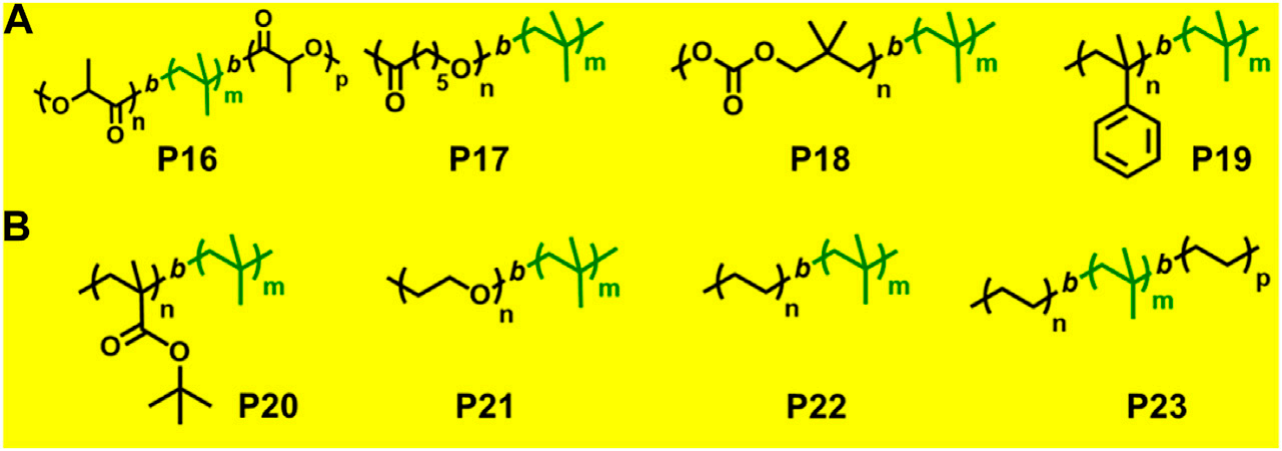
**(A)** Examples of block copolymers synthesized *via* combination of living cationic polymerization and ring-opening polymerization. **(B)** Examples of block copolymers synthesized *via* combination of living cationic polymerization with anionic polymerization and click chemistry.

ROP is a form of chain-growth polymerization, where the polymerization initiates through attack on cyclic monomer to form long chain polymers. Cyclic monomers such as epoxides, lactones, lactides, cyclic carbonates, and many strained cycloalkenes like norbornene undergo ROP with/without aid of metal catalyst. There are very few reports on synthesis of block copolymers by combined living cationic polymerization and ROP utilizing site transformation method (Figure 6A).

A few previously reported PIB based crystalline block copolymers are: poly(l-lactide-*b*-IB-*b*-l-lactide) (**P16**) triblock copolymer (Sipos et al., 1995), poly[IB-*b*-*ε*-caprolactone (*ε*-CL)] P(IB-*b*-*ε*-CL), (**P17**) diblock copolymer and poly(*ε*-CL-*b*-IB-*b*-*ε*-CL); Storey et al., 2001 triblock copolymers. Poly(l-lactide-*b*-IB-*b*-l-lactide) (**P16**) triblock copolymer was synthesized by ROP of l-lactide using α,ω-dihydroxy-polyisobutylene polymer as macroinitiator (Figure 5). Pivalolactone (PVL) and IB based block copolymer synthesis was accomplished by site transformation of living cationic polymerization of IB to anionic ring-opening polymerization (AROP) of PVL (Kwon et al., 2002). For the synthesis of P(IB-*b*-PVL) (**P18**) diblock copolymers; first, PIB with *ω*-carboxylate potassium salt was prepared by capping mechanism of living cationic PIB with DPE which was followed by quenching with 1-methoxy-1-trimethylsiloxy-propene (MTSP), and further hydrolysis of *ω*-methoxycarbonyl end groups to obtain the macroinitiator. Then, the *ω*-carboxylate potassium salt was used as a macroinitiator in tetrahydrofuran (THF) by the AROP process, to obtain P(IB-*b*-PVL) block copolymers. Similarly, P(PVL-*b*-IB-*b*-PVL) triblock copolymer was prepared by combined living cationic and AROP methodology as mentioned in the previous case, except for the difunctional initiator used for the polymerization of IB in the first step was taken as 5-*tert*-butyl-1,3-bis-(1-chloro-1-methylethyl)-benzene.

Novel glassy(A)-*b*-rubbery(B)-*b*-crystalline(C) linear triblock copolymers have been reported where glassy A block is poly(*α*-methylstyrene) P(*α*MeSt), rubbery B block is PIB, and crystalline C block is poly(PVL) (PPVL). The synthesis of P(*α*MeSt-*b*-IB) (**P19**) was accomplished by living cationic sequential polymerization followed by site transformation method using AROP to yield block copolymer of PVL. In the first synthetic step, the gel permeation chromatography (GPC) traces of P(*α*MeSt-*b*-IB) copolymers with *ω*-methoxycarbonyl functional group exhibited bimodal distribution in both refractive index (RI) and UV traces, and the small hump at higher elution volume was attributed to P*α*MeSt homopolymer. The homopolymer P*α*MeSt was removed to obtain the pure P(*α*MeSt-*b*-IB) macroinitiator by repeated fractionation using hexane/ethyl acetate and then utilized as macroinitiator for polymerization of PVL by AROP mechanism to produce P(*α*MeSt-*b*-IB-*b*-PVL) triblock copolymer. Complete crossover from living P*α*MeSt to IB is difficult. This was achieved modification of the living P(*α*MeSt) chain end with a small amount of *para*-chloro methylstyrene (*p*Cl*α*MeSt) after complete conversion of *α*MeSt. The poly(*α*MeSt-*b*-IB) copolymer carrying *ω*-carboxylate group, obtained from hydrolysis of *ω*-methoxycarbonyl group of the block copolymer, was used to initiate AROP of PVL in conjunction with 18-crown-6 in THF at 60°C, to produce P(*α*MeSt-*b*-*p*Cl*α*MeSt-*b*-IB-*b*-PVL) copolymer (Kwon and Faust, 2005).

Synthesis of poly(IB-*b*-ethylene oxide) diblock copolymer by combined living cationic and ROP has been reported by Groenewolt et al. (2005) Initially, HO-functional PIB was prepared by hydroboration/oxidation of allyl functional PIB, prepared from the reaction of living PIB and allyl trimethyl silane. The PIB alkoxide anion in conjunction with the bulky phospazene *t*-BuP_4_ is used as macroinitiator for ROP of ethylene oxide. Block copolymer of lactide (LA) and vinyl ether was reported, which showed thermo-responsiveness due to the poly(vinyl ether) segment. P(LA-*b*-vinyl ether) was precisely synthesized *via* successive living cationic polymerization of 2-methoxyethyl vinyl ether and ROP of lactide (Seki et al., 2018).

### Combination of Living Cationic Polymerization with Anionic Polymerization

The combination of living cationic and anionic techniques provides new scope for synthesis of block copolymers not obtainable by other methods. For example, IB and MMA monomers can be polymerized only by different mechanisms. P(IB-*b*-MMA) block copolymers were synthesized by coupling reaction of two corresponding living homopolymers, i.e., by living cationic and group transfer polymerization (GTP), respectively (Takács and Faust, 1995). Synthesis of P(MMA-*b*-IB-*b*-MMA) triblock copolymer has been reported using the site transformation method, using *α*,*ω*-dilithiated PIB as the macroinitiator (Kitayama et al., 1991; Kennedy et al., 1991). The key aspect is to cautiously control *α*—or *ω*-end functionality, as they are capable of initiating polymerization of the second monomer. Researchers have reported novel site transformation reaction by quantitative metalation of DPE-capped PIB carrying methoxy or olefin functional groups (Feldhusen et al., 1997). Metalation of DPE-capped PIB requires Na/K alloy as organolithium compounds. Polymers such as diblock P(IB-*b*-*t*BMA) (**P20**) and triblock P(MMA-*b*-IB-*b*-MMA) copolymers are also synthesized *via* this technique (Feldhusen et al., 1998). Another new synthetic route has been developed for the synthesis of P(IB-*b*-*t*BMA) by combining living cationic and anionic polymerizations, which involves metalation of 2-polyisobutylenyl-thiophene with *n*-butyllithium in THF at −40°C. The *t*BMA monomer was successfully polymerized using the synthesized stable macrocarbanion (PIB-T-Li^+^) initiator *via* living anionic polymerization, yielding P(IB-*b*-*t*BMA) block copolymers (Figure 5; Martinez-Castro et al., 2003). Similarly, synthesis of poly(IB-*b-*methyl methacrylate or hydroxyethyl methacrylate) block copolymers have also been reported by the combination of living cationic and anionic polymerization. DPE end-functionalized PIB (PIB-DPE) was prepared from the reaction of living PIB and 1,4-*bis*(1-phenyl ethenyl)benzene (PDDPE), and the resulting diphenyl carbenium ion was further methylated with dimethylzinc. The macroinitiator was generated by quantitative metalation of PIB-DPE with *n*-butyllithium in THF at room temperature. The final macroinitiator thus prepared could efficiently initiate the living polymerization of methacrylate monomers at –78°C to generate block copolymers with high block efficiency (Cho et al., 2006). Recently, Hadjichristidis et al. reported a series of hydroxyl-terminated poly(isobutylene-*b*-ethylene) P(IB-*b*-ethylene) (**P22**) copolymers using living cationic polymerization and polyhomologation (Zhang et al., 2016).

### Combination of Living Cationic Polymerization and Click Chemistry

Click coupling reactions, in particular copper(I)-catalyzed cycloadditions, have been successfully applied to prepare varieties of block copolymers. However, very few reports are there on synthesis of block copolymer by combination of living cationic polymerization and click chemistry. Diblock copolymer of azido functionalized PIB and alkyne terminated monomethyl ether was synthesized, using azido/alkene click chemistry in toluene solvent at 75°C to give PIB-*b*-PEO (**P21**) (Binder and Sachsenhofer, 2008). Similarly, azide/alkyne click reaction was used to synthesize three-armed star block copolymers of triazido-telechelic PIB and alkyne-terminated triethylene glycol monomethyl ether (Rother et al., 2010). The [1+3] cyclo-addition reaction between azido and alkene of azido end-functionalized polyethylenes and alkyne end-functionalized or telechelic PIBs respectively in a mixture of toluene and dimethylformamide at 110°C results in formation of di- and triblock copolymers P(IB-*b*-ethylene) (**P22**) and P(ethylene-IB-*b*-ethylene) (**P23**) (Espinosa et al., 2013). These new block copolymers are biocompatible and have potential application as thermoplastic elastomers.

### Combination of Cationic Polymerization with Other Polymerization Techniques

Living cationic ring-opening polymerization (CROP) of 2-substituted 2-oxazoline provides new scope for synthesis of different architectures. Zhang et al. (2009) reported graft copolymer using dual functional monomer, 2-isopropenyl-2-oxazoline. First, the monomer undergoes free radical or living anionic polymerization to form the backbone, which is converted to macroinitiator salt for CROP of 2-Oxazolines. These grafted polymers show molecular brush architecture which can further be applied in biomedical fields. CROP of 2-Oxazolines may be carried out by microwave-assisted heating (Petit et al., 2017). Recently, Zhu et al. (2000) reported free-radical-promoted cationic polymerization of cyclohexene oxide, *n*-butyl vinyl ether, and N-vinyl carbazole under chemiluminescence irradiation.

## Conclusions and Perspectives

Presently, design and synthesis of polymers with well-defined complex architectures having advanced properties is an important field of research in polymer science for varied industrial applications. Numerous reports are present for the synthesis of functional copolymers with varied architectures like block, graft, star, and brush by simple controlled living polymerizations; but are limited to monomers polymerizable by the same polymerization mechanism and on their relative monomer reactivity. In this review we have summarized recent advancements in synthetic processes of novel block copolymers with well-known monomers by combining different polymerization mechanisms like anionic, ATRP, RAFT, NMP, and ROP with living cationic polymerization. Thus, this review reveals many innovative breakthroughs and huge developments in the area of block copolymer synthesis by the combination of living cationic polymerization and other polymerization methods.

Synthesis of new polymeric architectures by combining two techniques is quite challenging. By the discussed approaches a whole new range of copolymers can be prepared that were not available by polymer preparation using only coupling or sequential monomer addition. Copolymers may be synthesized by site transformation reaction from one polymerization technique to another or by applying a dual functional initiator capable of polymerizing two types of monomers by two different techniques. These approaches open a new avenue in the field of complex macromolecular architecture development. Up to now, site transformation reactions and synthesis of new dual initiators have made tremendous progress. The investigation of structurally well-defined polymers and their synthetic procedure will remain a hot topic to pursue, in both industry and academia.
